# Attempted retrieval of guidewire fragment using the twisting wire technique causes coronary perfusion: Case report

**DOI:** 10.1097/MD.0000000000037842

**Published:** 2024-04-19

**Authors:** Zi-Wei Zhang, Xiao-Juan Pan, Ji-Hong Zou, Feng Qi

**Affiliations:** Department of center for coronary heart disease, Fu Wai Yunan Cardiovascular Hospital, Kunming, China.

**Keywords:** contralateral angiography, coronary perforation, guidewire fracture, percutaneous coronary intervention, twisting wire technique

## Abstract

**Rationale:**

Guidewire fracture is one of the biggest risks of percutaneous coronary intervention, twisting wire technique is very useful for retrieving the fractured wire, but the potential risks have been inadequately reported. Herein, we present a case of retrieval of guidewire fragments using the twisting wire technique that causes coronary perfusion.

**Patient concerns:**

A 37-year-old male patient was admitted to our hospital for elective percutaneous coronary intervention of the left circumflex coronary artery.

**Clinical findings:**

For chronic total occlusion of the distal left circumflex coronary artery, antegrade recanalization by wire escalation, and parallel wire techniques were attempted. However, we shockingly found that the BMW guidewire, anchored in the right coronary artery, spontaneously fractured from the proximal right coronary artery, and a lengthy fragment of the guidewire remained in the coronary.

**Diagnoses, interventions, and outcomes:**

Many attempts were made to retrieve the remnant guidewire including the twisting wire technique, which leads to the perforation of the coronary.

**Outcomes:**

Finally, percutaneous retrieving procedures were stopped in favor of surgical extraction via a small coronary arteriotomy. This procedure was successful.

**Lessons:**

To the best of our knowledge, the present case is the first reported spontaneous fracture of the guidewire. Leaving such a lengthy remnant guidewire in the artery, or leaving stenting over the wire, would impose a high risk of coronary thrombosis, perforation, and embolization. Yet, the perforation of the artery that occurred in this case, which could have had life-threatening consequences, resulted from our attempts to retrieve the guidewire using the twisting wire technique.

## 1. Introduction

A guidewire that breaks inside the coronary artery during an intervention can cause immediate complications as well as adverse events over time. It must be removed. Retrieval methods to extract a guidewire fragment include deep engagement of the guiding catheter or techniques such as the loop snare or twisting wire. The latter used to retrieve the fragment threatens coronary perforation, as the broken end of the guidewire is hard and sharp and can easily penetrate the coronary artery wall.

In the present case, several pullback maneuvers using the twisting wire technique were performed to withdraw a guidewire fragment from the ostial right coronary artery (RCA), but coronary perforation occurred. When all attempts at percutaneous retrieval failed, surgical removal was necessary and successful. The patient had an uneventful recovery without signs or complications, as of the 3-month follow-up.

We conclude that fracture of the guidewire remains a dangerous and unpredictable complication of percutaneous coronary intervention, and we especially highlight the potential risk of the twisting wire technique when used for retrieving the fractured wire from the coronary artery. Although the twisting wire technique is suitable for withdrawing a fractured guidewire in selected cases, it must be used with great caution. For our patient, not only did the twisting wire technique fail to retrieve the broken guidewire fragment, but a most serious consequence was the penetration of the wall of the coronary artery.

## 2. Case presentation

**Figure 1.**
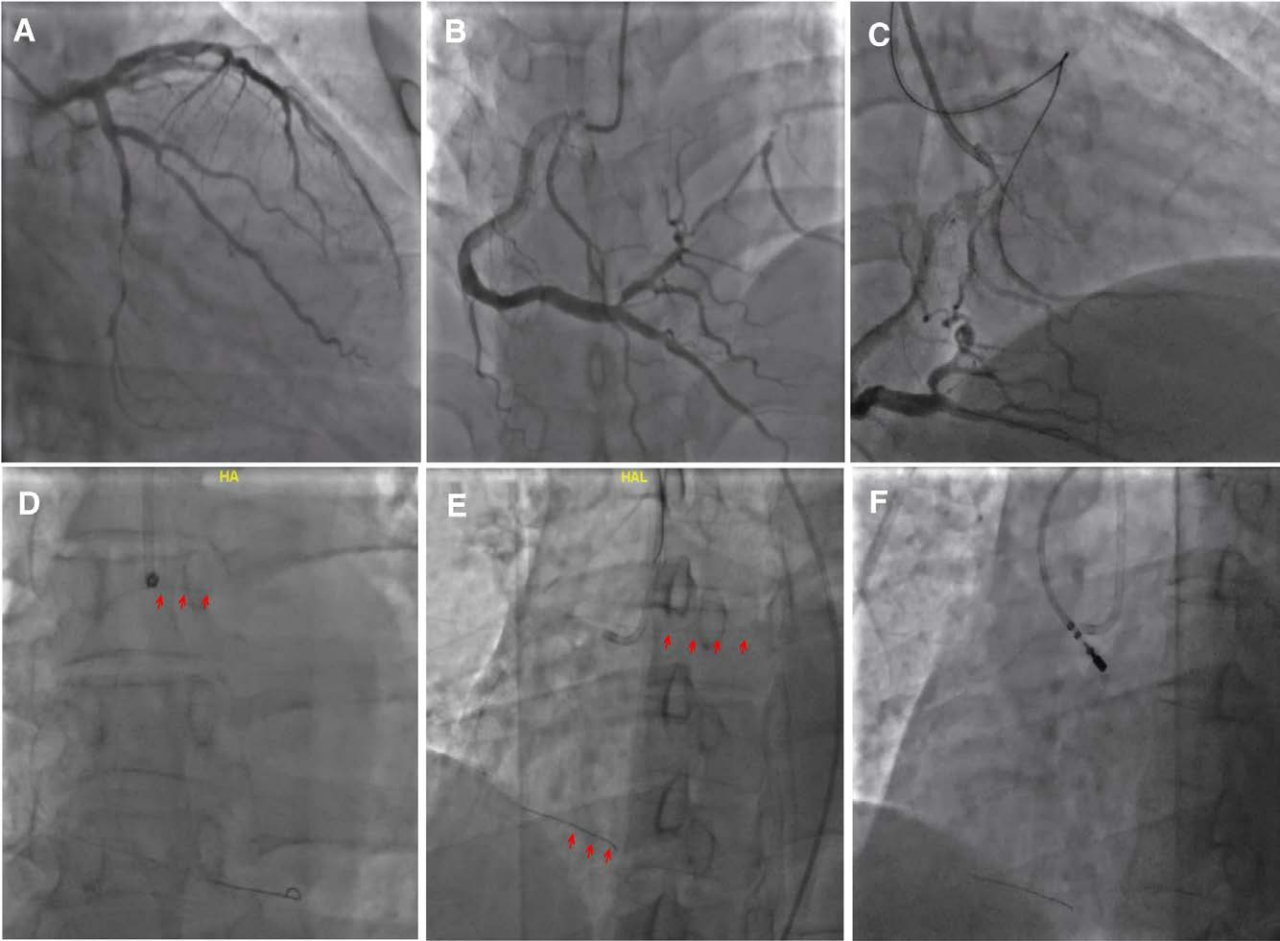
Coronary angiograms during percutaneous coronary intervention. (A) Total occlusion of the distal left circumflex artery. (B) The RCA supplied a well-developed collateral circulation to the left circumflex artery. (C) Attempts at passage of an antegrade wire using wire escalation and parallel wire techniques under the guidance of the contralateral angiogram. (D) The guidewire spontaneously fractured and anchored in the RCA (red arrows). (E) Withdrawal of the fractured guidewire via the twisting wire technique (red arrows). (F) A goose-neck snare failed to capture the proximal end of the remnant wire even when assisted by a steering coronary sinus catheter at the site of the aorta sinus. RCA = right coronary artery.

A 37-year-old male patient was admitted to our hospital for elective percutaneous coronary intervention of the left circumflex coronary artery. The man had a history of acute inferior myocardial infarction with recurrent effort angina class II and had undergone emergency stenting in the RCA in the previous month (Fig. 1A and B).

A 0.014-inch BMW (Balance Middleweight) guidewire (Abbott Vascular, Santa Clara, CA, USA) was inserted into the distal RCA as an anchor wire to stabilize the 7Fr JR4 guiding catheter used for contralateral angiography (Fig. 1C). For chronic total occlusion of the distal left circumflex coronary artery, antegrade recanalization by wire escalation and parallel wire techniques were attempted. However, we shockingly found that the BMW guidewire, anchored in the RCA, spontaneously fractured from the proximal RCA, and a lengthy fragment of the guidewire remained in the coronary (Fig. 1D).

Spontaneous fracture of the guidewire during contralateral angiography is exceptionally rare, and reports of accidental coronary perforation resulting from retrieval attempts during catheterization are few. Factors that can cause this complication include improper placement of the guidewire, damage from the guiding catheter to the guidewire, polymer damage within the implanted stent, forceful withdrawal of the wire, manufacturing defects, or any combination of these.^[1]^

In the present case, we initially attempted to reengage the 7Fr JR4 guiding catheter over the fractured wire and then inflated the balloon inside the guiding catheter to trap it. In this way, we might retrieve the remnant wire by simultaneous withdrawal of all the devices. Unfortunately, repeated attempts to engage the JR4 guiding catheter over the fractured wire were futile, as the broken tip of the wire may have become firmly attached or fixed to the coronary artery wall.

Three Runthrough 0.014-inch guidewires (Terumo) were sequentially introduced into the distal part of the RCA and rotated simultaneously within a torque, and the guidewire fragment became entangled with them. We tried to pull all the entangled wires simultaneously into the guiding catheter, but the recalcitrant wire appeared to move from the ostial RCA to the aorta sinus (Fig. 1E).

Then many attempts were made to retrieve the remnant guidewire at the aorta sinus using the snare loop technique. A goose-neck snare was inserted into the aorta sinus via the left radial artery, but this failed to capture the offending wire. A steerable coronary sinus catheter was also manipulated to change the location of the guidewire and make capturing with the snare easier (Fig. 1F), but all these attempts failed.

Other options used were a self-made wire snare or retrievable stent system (Solitare) to grab the distal end of the guidewire. However, the proximal end of the fractured wire was hard and sharp, and may already have perforated the artery wall when the twisting wire technique was attempted to pull the remnant back.

**Figure 2.**
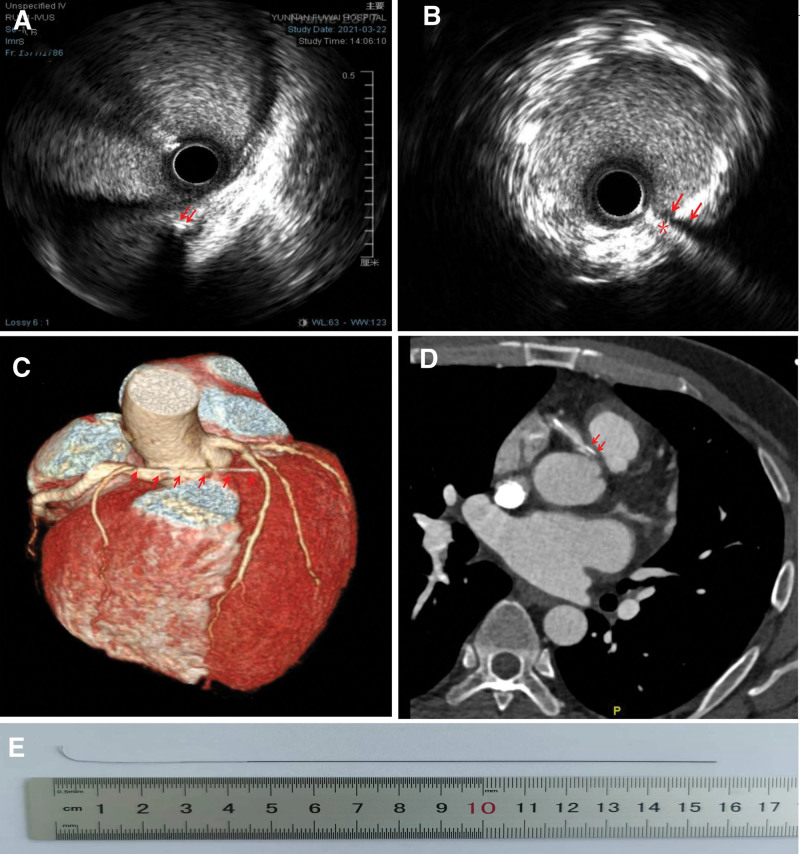
(A) The IVUS revealed that the fractured guidewire had penetrated the adventitia of the proximal RCA (red arrow). (B) The mid-segment of the guidewire was lodged by the previously implanted stent (red arrow). (C) CTA, axial view. The proximal end of the guidewire had already perforated the RCA wall (red arrow). (D) The CTA showed that the guidewire had penetrated the anterior wall of the left ventricle (red arrow). (E) The 16-cm broken BMW wire fragment was removed surgically. IVUS = intravascular ultrasound; RCA = right coronary artery.

Intravascular ultrasound (IVUS) usually enables us to detect if a broken wire has perforated the proximal RCA, but this was prevented by the previously implanted stent. The maneuvers to forcefully pull back the entangled wires may have deformed the deployed stent and it was very difficult to pass the IVUS catheter through it. Finally, the IVUS catheter was successfully advanced to the distal RCA after several high-pressure balloon dilatation procedures. It was shown that the broken guidewire had already perforated the adventitia of the proximal RCA (Fig. 2A) and the mid-segment of the wire was stuck because of the stent (Fig. 2B). If we persisted in using other percutaneous retrieval techniques to snare the distal end of the guidewire, the consequences were potentially dire or life-threatening. Subsequent coronary computed tomography angiography also showed that the broken guidewire had penetrated the pericardium, reaching the front wall of the left ventricle (Fig. 2C and D).

Finally, percutaneous retrieving procedures were stopped in favor of surgical extraction via a small coronary arteriotomy. This procedure was successful (Fig. 2E).

## 3. Discussion and conclusions

The development of new devices and techniques in percutaneous revascularization of coronary artery diseases has allowed dramatic improvements in the outcomes of increasingly complex interventions. However, these procedures remain challenging and one of the risks is guidewire fracture, with an incidence of 0.1% to 0.2%. A guidewire may break if trapped by a stent strut, or if over-rotated,^[2]^ by forceful manipulations in cases of severe calcification or tortuous lesion,^[3]^ or, rarely, due to flaws in manufacture. The recommended management of complications associated with percutaneous coronary intervention depends on whether the guidewire is intact, has exited the artery, or is still in the guide.^[4]^

To the best of our knowledge, the present case is the first reported spontaneous fracture of the guidewire. Leaving such a lengthy remnant guidewire in the artery, or leaving stenting over the wire, would impose a high risk of coronary thrombosis, perforation, and embolization. Yet, the perforation of the artery that occurred in this case, which could have had life-threatening consequences, resulted from our attempts to retrieve the guidewire using the twisting wire technique.

**Figure 3.**
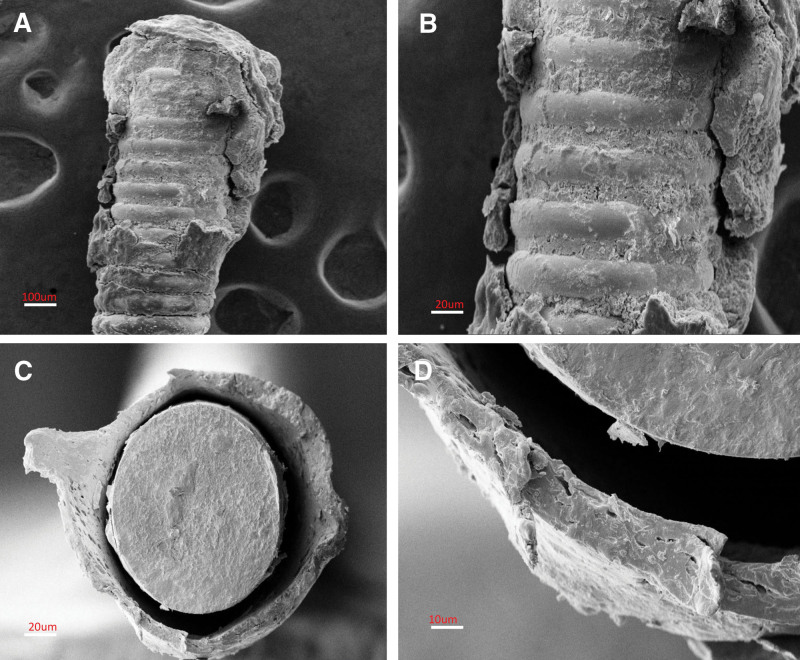
(A and B) Scanning electron microscopy showed the compromised integrity of the guidewire coatings due to damage caused by the twisting wire technique. (C and D) Scanning electron microscopy, axial view of the proximal end of the guidewire. The polymer coatings were completely separated from the metal coil surfaces.

The probability of guidewire fracture may be higher during procedures that involve a complex lesion, but spontaneous fracture during contralateral angiography is relatively unexpected. In the present case, the design of the BMW guidewire may have facilitated the breakage. Under a scanning electron microscope, there appeared obvious damage to the coating of the distal segment of the broken guidewire, due to twisting the wire during the attempted retrieval; the polymer coating of the proximal end was entirely separated from the metal coil surface, while the surface of the fracture was very flat and smooth (Fig. 3). This suggests a problem at the junction of the guidewire and shaft.

In this case, the BMW wire was inserted into the distal RCA to act as an anchor wire to stabilize the guiding catheter. From the view of the angiography, the engaged noncoaxial guiding catheter may have placed the guidewire at an acute angle, so that the tip of the 7Fr JR4 guiding catheter, with the cardiac motion, repeatedly hit against the guidewire. IVUS also showed that the mid-segment of the wire was trapped by the previously deployed stent. The constriction of the wire against the guiding catheter may have triggered the fracture. There was too much force imposed at the fracture site due to the severe deformation of the anchored wire within the deployed stent. In addition, the noncoaxial guiding catheter damaged the wire, leading ultimately to the fracture of the guidewire’s polymer coating and core.

Although the twisting wire technique is very effective and useful, the potential risks have been inadequately reported. These include deformation by the deployed stent, coronary perforation, polymer damage that could result in myocardial infarction,^[5]^ secondary fracture, and possible distal vessel dissection. The challenges and risks are comparable to other percutaneously retrieving procedures. Ultimately, in our case, the emergency surgical extraction of the retained guidewire fragment was successful.

Safer methods such as improvements in the guidewire to prevent breakage and methods of retrieval are needed and would be a major breakthrough in this field.

## Author contributions

**Conceptualization:** Zi-Wei Zhang, Ji-Hong Zou.

**Data curation:** Zi-Wei Zhang, Feng Qi.

**Investigation:** Zi-Wei Zhang, Ji-Hong Zou.

**Software:** Zi-Wei Zhang.

**Writing—original draft:** Zi-Wei Zhang, Xiao-Juan Pan, Ji-Hong Zou, Feng Qi.

**Funding acquisition:** Xiao-Juan Pan.

**Project administration:** Xiao-Juan Pan, Feng Qi.

**Visualization:** Ji-Hong Zou.
